# Biogenesis and regulation of the *let-7* miRNAs and their functional implications

**DOI:** 10.1007/s13238-015-0212-y

**Published:** 2015-09-23

**Authors:** Hosuk Lee, Sungwook Han, Chang Seob Kwon, Daeyoup Lee

**Affiliations:** Department of Biological Sciences, Korea Advanced Institute of Science and Technology, Daejeon, 305-701 Korea; Department of Chemistry and Biology, Korea Science Academy of KAIST, Busan, 614-822 Korea

**Keywords:** miRNA processing, miRNA biogenesis, let-7 family, TUTase, LIN28A/B

## Abstract

The *let-7* miRNA was one of the first miRNAs discovered in the nematode, *Caenorhabditis elegans*, and its biological functions show a high level of evolutionary conservation from the nematode to the human. Unlike in *C. elegans*, higher animals have multiple isoforms of *let-7* miRNAs; these isoforms share a consensus sequence called the ‘seed sequence’ and these isoforms are categorized into *let-7* miRNA family. The expression of *let-7* family is required for developmental timing and tumor suppressor function, but must be suppressed for the self-renewal of stem cells. Therefore, *let-7* miRNA biogenesis must be carefully controlled. To generate a *let-7* miRNA, a primary transcript is produced by RNA polymerase II and then subsequently processed by Drosha/DGCR8, TUTase, and Dicer. Because dysregulation of *let-7* processing is deleterious, biogenesis of *let-7* is tightly regulated by cellular factors, such as the RNA binding proteins, LIN28A/B and DIS3L2. In this review, we discuss the biological functions and biogenesis of *let-7* miRNAs, focusing on the molecular mechanisms of regulation of *let-7* biogenesis in vertebrates, such as the mouse and the human.

## INTRODUCTION

MicroRNAs (miRNAs) are short (~22-nucleotide-long) non-coding RNAs found in diverse eukaryotes from plants to animals. They inhibit gene expression largely in a post-transcriptional manner, by recognizing a specific complementary sequence usually located in the 3′ UTR of a target mRNA. The binding of a miRNA to this complementary sequence decreases translation of the target mRNA via several mechanisms, including mRNA degradation, inhibition of translational initiation and elongation (Eulalio et al., 2008; Filipowicz et al., 2008; Ameres and Zamore, 2013; Ha and Kim, 2014).

*Let-7* (*lethal-7*) was one of the first miRNAs to be discovered. It was originally identified as a regulator of developmental timing in the nematode, *C. elegans*, and was therefore regarded as a heterochronic gene (Reinhart et al., 2000). The *let-7* miRNA is evolutionarily conserved across various animal species, including flies and mammals, but it is not found in plants (Pasquinelli et al., 2000; Hertel et al., 2012). The nematode and fruit fly have a single isoform, whereas higher animals have multiple *let-7* isoforms. In the human, for instance, the *let-7* family is composed of nine mature *let-7* miRNAs encoded by 12 different genomic loci, some of which are clustered together (Ruby et al., 2006; Roush and Slack, 2008).

As *let-7* expression gradually increases during development, and this miRNA plays important roles in many biological processes, it could be expected that the biogenesis of *let-7* should be tightly regulated (Pasquinelli et al., 2000; Sempere et al., 2002; Thomson et al., 2006; Liu et al., 2007). Indeed, studies have shown that LIN28A/B blocks *let-7* biogenesis in several different ways to maintain self-renewal and pluripotency in stem cells (Heo et al., 2008; Newman et al., 2008; Rybak et al., 2008; Viswanathan et al., 2008; Heo et al., 2009; Piskounova et al., 2011; Kim et al., 2014). In addition, TUTase has been shown to be involved in degrading the *let-7* precursor (*pre-let-7*) to block the generation of mature *let-7* in the cytoplasm (Hagan et al., 2009; Heo et al., 2009; Thornton et al., 2012).

In this review, we briefly summarize the current state of knowledge regarding the *let-7* miRNA family and its biological functions, focusing on *let-7* biogenesis in higher animals. In addition, we discuss recent progress in better understanding the regulatory mechanisms that act upon *let-7*.

## GENERAL FEATURES OF THE *let-7* FAMILY

### The discovery of *let-7* in *C. elegans*

**Figure 1:**
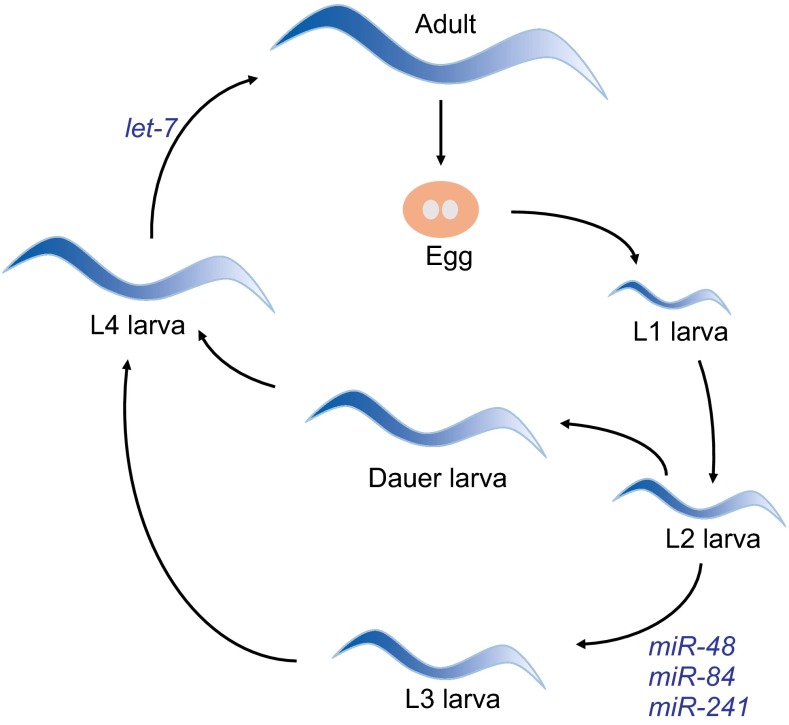
**Life cycle of the nematode,** ***Caenorhabditis elegans***. Schematic diagrams of the *C. elegans* life cycle. Eggs laid by adult *C. elegans* go through four developmental stages: L1, L2, L3, and L4 larva. If the environment is harsh, L2 larva can go through the Dauer larva stage instead of the L3 larva stage. During the life cycle of *C. elegans*, *miR-48*, *miR-84*, and *miR-241* regulate the L2-to-L3 transition, whereas *let-7* regulates the L4-to-adult transition

Experiments using forward genetics originally identified *let-7* (*lethal-7*) as a heterochronic gene in *C. elegans* (Reinhart et al., 2000). Heterochronic genes act sequentially to regulate cell fates in a stage-specific manner during the different larval transitions in *C. elegans* (Moss, 2007). For instance, *miR-48*, *miR-84*, and *miR-241* regulate the second larval (L2) to third larval (L3) transition, while *let-7* regulates the fourth larval (L4) to adult transition (Fig. 1) (Reinhart et al., 2000; Abbott et al., 2005). During the development of *C. elegans*, hypodermal seam cells undergo asymmetric division in a manner similar to that seen in stem cells. As a result, one daughter cell undergoes differentiation, while the other undergoes self-renewal at each larval stage. At the final transition (the L4-to-adult transition), all of the daughter cells stop proliferation and undergo differentiation. After this terminal differentiation, the seam cells form alae. In contrast, seam cells harboring the *let-7* mutation fail to finish the L4-to-adult transition and instead exhibit extra cell division without proper formation of the adult alae (Reinhart et al., 2000). As a result, the majority of *let-7* mutants die due to bursting of the vulva, earning this mutation its name: *lethal-7*. The expression pattern of *let-7* is consistent with its mutant phenotype, as its expression is first detected at the L3 stage and peaks at the L4 stage (Reinhart et al., 2000; Esquela-Kerscher et al., 2005). In addition, precocious expression of *let-7* at the L2 stage yielded an early adult-like phenotype at the L4 stage (Hayes and Ruvkun, 2006). These studies collectively support the notion that *let-7* is a key regulator of proper developmental timing in *C. elegans*.

### Characteristics of the *let-7* family

**Figure 2:**
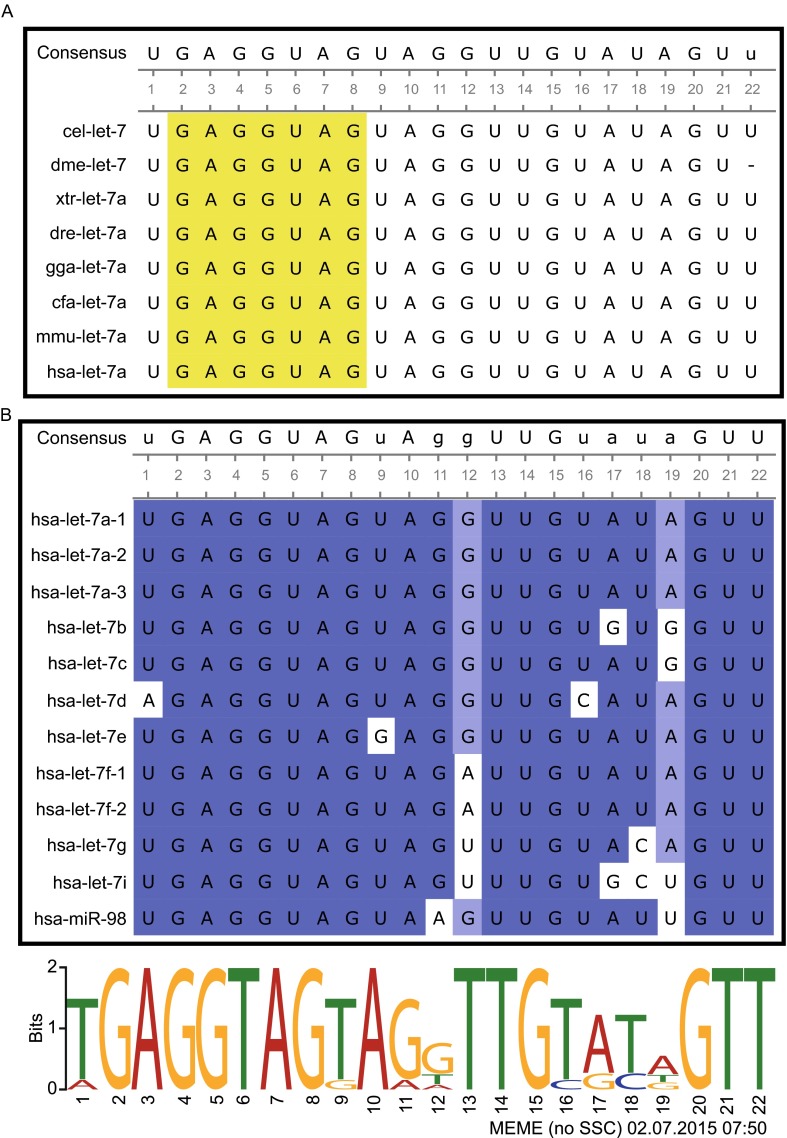
**Sequence comparison of** ***let-7*** **family members across diverse animal species**. (A) *C. elegans* (cel), *D. melanogaster* (dme), *X. tropicalis* (xtr), *D. rerio* (dre), *G. gallus* (gga), *C. familiaris* (cfa), *M. musculus* (mmu), and *H. sapiens* (hsa) all possess the consensus mature *let-7* (*let-7a*) sequence of ‘UGAGGUAGUAGGUUGUAUAGUU’. The seed sequence is indicated as a yellow box. Consensus mature sequences are placed at the top of the box, where only perfectly aligned sequences are capitalized. (B) Sequence alignment of the mature forms of human *let-7* family members (upper panel). Dark blue box represents percentage identity over 70%, whereas light blue box indicates percentage of over 50%. Consensus mature sequences are placed at the top of the box, where only perfectly aligned sequences are capitalized. Consensus sequences of the mature human *let-7* family members, as assessed by MEME (http://meme-suite.org, bottom panel)

*Let-7* miRNAs are found in various animal species, including the human. This conservation suggests that *let-7* may act as a regulator of gene expression across diverse animal species (Pasquinelli et al., 2000; Hertel et al., 2012). Using computational analyses, such as BLAST (Basic Local Alignment Search Tool), researchers have discovered a total of 28,645 miRNAs from 223 species that have been recorded in miRBase release 21.0 (http://www.mirbase.org). This substantial total includes 401 *let-7* sequences from various organisms. According to miRBase, *Caenorhabditis elegans* (nematode), *Drosophila melanogaster* (fly), *Xenopus tropicalis* (frog*), Danio rerio* (zebra fish), *Gallus gallus* (chicken), *Canis familiaris* (dog), *Mus musculus* (mouse) and *Homo sapiens* (human) all express a version of *let-7* (*let-7a*) that possesses the exact consensus sequence of ‘UGAGGUAGUAGGUUGUAUAGUU’ (Fig. 2A). Most of *let-7* sequences include the ‘seed sequence’. This highly preserved sequence that spans nucleotides 2 through 8 in some miRNAs (Ruby et al., 2006), and is an essential component required for target recognition by the RNA-induced silencing complex (RISC) (Brennecke et al., 2005; Grimson et al., 2007; Hibio et al., 2012). This conserved feature of the *let-7* miRNAs suggests that their targets and functions may be similar across diverse animal species.

Although the *let-7* sequence is well conserved from the nematode to the human, several differences distinguish the closely related *let-7* family members of various animal species (Roush and Slack, 2008). For one, whereas the nematode and the fly have only one *let-7* miRNA, higher animals (e.g., fishes and mammals) have diverse *let-7* family members including *let-7a*, -*7b*, -*7c*, -*7d*, -*7e*, -*7f*, -*7g*, -*7h*, -*7i*, -*7j*, -*7k* (see below for a discussion of this nomenclature) and *miR-98* (Table 1) (Lagos-Quintana et al., 2001; Lau et al., 2001; Chen et al., 2005; Landgraf et al., 2007). Higher animals have generally similar sets of *let-7* family members, although slight differences may be observed (for example, *let-7h* exists in the zebrafish but not in the human). Notably, each *let-7* family member is often present in multiple copies across the genomes of higher animals (Table 1). To distinguish between the various isoforms, a letter and/or number are placed after the term ‘*let-7*’. Sequence differences are indicated by letters (e.g., *let-7a* and -*7b*), while different genomic loci expressing the same sequence are indicated by numbers. As an example of the latter, the precursors (also known as the stem-loop sequence in miRBase) of human *let-7a-1*, *let-7a-2*, and *let-7a-3* are encoded on chromosomes 9, 11, and 12, respectively, but all produce the same *let-7a* miRNA (Fig. 2B and Table 1). Thus, the numbers of precursor sequences encoded in the genome of a given species may differ from the number of mature miRNAs expressed in that species. In the human, for example, 12 distinct loci encode nine mature *let-7* miRNAs (Fig. 2B and Table 2).

**Table 1:** Characterization of *let-7* family across different species

Mature let-7	*C. elegans* nematode	*D. melanogaster* fly	*D. rerio* zebrafish	*M. musculus* mouse	*H. sapiens* human
*let-7a*	*let-7*	*let-7*	*let-7a-1*, *2*, *3*, *4*, *5*, *6*	*let-7a-1*, *2*	*let-7a-1*, *2*, *3*
*let-7b*			*let-7b*	*let-7b*	*let-7b*
*let-7c*			*let-7c-1*, *2*	*let-7c-1*, *2*	*let-7c*
*let-7d*			*let-7d-1*, *2*	*let-7d*	*let-7d*
*let-7e*			*let-7e*	*let-7e*	*let-7e*
*let-7f*			*let-7f*	*let-7f-1*, *2*	*let-7f-1*, *2*
*let-7g*			*let-7g-1*, *2*	*let-7g*	*let-7g*
*let-7h*			*let-7h*		
*let-7i*			*let-7i*	*let-7i*	*let-7i*
*let-7j*			*let-7j*		
*let-7k*					
*miR-98*				*miR-98*	*miR-98*

Mature *let-7* family members (*let-7a*, -*7b*, -*7c*, -*7d*, -*7e*, -*7f*, -*7g*, -*7h*, -*7i*, -*7j*, -*7k*, and *miR-98*) and their corresponding precursors in *Caenorhabditis elegans*, *Drosophila melanogaster*, *Danio rerio*, *Mus musculus*, and *Homo sapiens* are presented

**Table 2:** Genomic location and conserved clusters of *let-7* family in human and fly

*let-7* Family	Genome context	Clusters
*H. sapiens*		
*hsa-let-7a-2*	chr11: 122146522-122146593 −	Cluster1-a (*let-7a-2*, *miR-100*, *miR-125b-1*)
*hsa-let-7c*	chr21: 16539828-16539911 +	Cluster1-b (*let-7c*, *miR-99a*, *miR-125b-2*)
*hsa-let-7e*	chr19: 51692786-51692864 +	Cluster1-c (*let-7e*, *miR-99b*, *miR-125a*)
*hsa-let-7a-1*	chr9: 94175957-94176036 +	Cluster2 (*let-7a-1*, *-7d*, *-7f-1*)
*hsa-let-7d*	chr9: 94178834-94178920 +	
*hsa-let-7f-1*	chr9: 94176347-94176433 +	
*hsa-let-7a-3*	chr22: 46112749-46112822 +	Cluster3 (*let-7a-3*, *-7b*)
*hsa-let-7b*	chr22: 46113686-46113768 +	
*hsa-let-7f-2*	chrX: 53557192-53557274 −	Cluster4 (*let-7f-2*, *miR-98*)
*hsa-miR-98*	chrX: 53556223-53556341 −	
*hsa-let-7g*	chr3: 52268278-52268361 −	
*hsa-let-7i*	chr12: 62603686-62603769 +	
*D. melanogaster*		
*dme-let-7a-2*	2L: 18472034-18472111 +	Cluster1 (*let-7*, *miR-100*, *miR-125*)

Precursors of human and fly *let-7* family can be located individually (*let-7g*, *-7i*) or as clusters (cluster 1 to 4). Genomic location and four clusters of these precursors are described

In animal genomes, the *let-7* family members can be encoded individually or as clusters with other family members and/or unrelated miRNAs. Comparison of *let-7* family members in *D. melanogaster* and higher animals has revealed that such sequences tend to show similar genomic positions, suggesting that they form well-preserved clusters (Lagos-Quintana et al., 2001; Bashirullah et al., 2003; Sempere et al., 2003). In the human, *let-7g* and *let-7i* are located individually on chromosomes 3 and 12, respectively, while the other *let-7* family members are distributed among four clusters (clusters 1 to 4) (Table 2). Cluster 1, which contains three miRNAs, including *let-7a*, *miR-100*, and *miR-125*, is also conserved in *D. melanogaster* (Table 2). Cluster 1 can be further sub-classified into three clusters (cluster 1-a, 1-b, and 1-c) by its location and components. Interestingly, cluster 1-a and cluster 1-b are involved in hematopoietic stem and progenitor cell (HSPC) homeostasis by regulating the balance between TGFβ and Wnt signaling (Emmrich et al., 2014), whereas cluster 1-c is highly expressed in HSPC and confers hematopoietic phenotypes (Gerrits et al., 2012). However, *miR-125a* is responsible for most of these properties in cluster 1-c and the transcription of miRNAs in cluster 1-a (*let-7a-2*, *miR-100*, and *miR-125b-1*) are loosely related (Sempere et al., 2004; Gerrits et al., 2012). Cluster 2 contains *let-7a*, *-7d*, and *-7f-1*, whereas cluster 3 is composed of *let-7a-3* and *-7b*. Lastly, cluster 4 is consisted of *let-7f-2* and *miR-98* (Table 2). Vertebrate-specific genomic duplication events are thought to be responsible for the formation of these clusters (Hertel et al., 2012), which may confer proper regulation and correct biogenesis of the involved miRNAs.

### Biological roles of *let-7* family members

The high degree of conservation among *let-7* miRNAs across different animal species suggests that they may play important (and potentially similar) roles in the biological processes of various organisms (Pasquinelli et al., 2000; Hertel et al., 2012). Indeed, recent studies have shown that *let-7* family members generally promote differentiation during development and function as tumor suppressors in various cancers (Reinhart et al., 2000; Takamizawa et al., 2004; Grosshans et al., 2005; Johnson et al., 2005; Yu et al., 2007; Caygill and Johnston, 2008; Kumar et al., 2008).

In *C. elegans*, *let-7* controls the crucial developmental timing of the last larval transition (L4-to-adult) via regulation of transcription factors (*daf-12*, *pha-4*, *die1*, and *lss4*) in different tissues (Fig. 1) (Reinhart et al., 2000; Grosshans et al., 2005). *let-7* has also been shown to function as a heterochronic gene in *D. melanogaster* (Caygill and Johnston, 2008; Sokol et al., 2008), wherein *let-7* mutants show abnormal (delayed) cell cycle exit in the wing (Caygill and Johnston, 2008) and an irregular maturation of neuromuscular junctions in the adult abdominal muscles that results in immaturity of the neuromusculature and defects in adult fertility, motility, and flight (Sokol et al., 2008). Consistent with this mutant phenotype, *let-7* expression in *D. melanogaster* gradually increases during the third larval instar stage and peaks in the pupa (Pasquinelli et al., 2000; Bashirullah et al., 2003). Thus, the *let-7* miRNAs of *C. elegans* and *D. melanogaster* both act as essential regulators for proper development at the larva-to-adult transition. In chicken and mice, *let-7* is involved in limb development (Mansfield et al., 2004; Lancman et al., 2005; Schulman et al., 2005).

In mammals, *let-7* expression is high during embryogenesis and brain development (Thomson et al., 2004; Schulman et al., 2005; Thomson et al., 2006; Wulczyn et al., 2007) and remains high in adult tissues (Sempere et al., 2004; Thomson et al., 2004). Moreover, *let-7* is known to regulate hematopoietic stem cell fate along with *miR-99a/100*, *miR-125b-1/2*, and LIN28B (Copley et al., 2013; Lee et al., 2013b; Emmrich et al., 2014). Cluster1-a (*let-7a-2*, *miR-100*, *miR-125b-1*) and Cluster1-b (*let-7c*, *miR-99a*, *miR-125b-2*) are involved in HSPC (hematopoietic stem and progenitor cell) homeostasis such as self-renewal, proliferation, quiescence, and differentiation by blocking TGFβ pathway and amplifying Wnt signaling (Emmrich et al., 2014), whereas LIN28B represses *let-7* to inhibit erythroid development and maintain stemness (Copley et al., 2013; Lee et al., 2013b). However, the exact role of *let-7* family members in mammalian development has not yet been fully elucidated (Lancman et al., 2005; Schulman et al., 2005; Wulczyn et al., 2007), in large part because it is technically difficult to knock out multiple *let-7* family members in the same individual. Moreover, these multiple *let-7* family members are likely to have functionally redundant roles.

With respect to the function of *let-7* as tumor suppressor, the targets of *C. elegans let-7* were initially predicted using computational analysis, and the 3′ UTR of *let-60* [also known as an ortholog of the RAS (human Rat sarcoma) oncogene] was identified as having the highest identified sequence complementarity to *let-7* (Johnson et al., 2005). Subsequently, *let-7* was shown to interact with *let-60* and RAS in *C. elegans* and human cancers, respectively (Johnson et al., 2005). Moreover, up-regulation of RAS was found to require down-regulation of *let-7* in lung cancer and non-small cell lung cancer (NSCLC) (Takamizawa et al., 2004; Johnson et al., 2005; Kumar et al., 2008), and *let-7g* was shown to block tumorigenesis by suppressing RAS in NSCLC (Kumar et al., 2008). In addition to the role of *let-7* in modulating the RAS oncogene, multiple *let-7* members were found to be down-regulated in human cancers and cancer stem cells, strengthening the notion that *let-7* may also function as a tumor suppressor (Takamizawa et al., 2004; Shell et al., 2007; Yu et al., 2007; Dahiya et al., 2008; O’Hara et al., 2009). Several other lines of evidence strongly suggest that *let-7* functions as tumor suppressor in general. For example, *let-7* family members have been shown to repress cell cycle regulators (e.g., cyclin A, cyclin D1, cyclin D3, and CDK4) and block cell cycle progression and anchorage-independent growth in cancer cells (Johnson et al., 2007; Schultz et al., 2008). Additionally, *let-7a* reportedly inhibits MYC-induced cell growth in Burkitt lymphoma cells by blocking MYC expression (Sampson et al., 2007). Moreover, HuR, RNA-binding protein, binds and represses MYC mRNA by recruiting the *let-7*/RISC complex to 3′ UTR region of MYC (Ma et al., 1996; Kim et al., 2009). In addition, recruitment of HuR and *let-7* to the transcript of MYC is interdependent (Kim et al., 2009; Gunzburg et al., 2015). Interestingly, MYC can also negatively regulate *let-7* family members such as *let-7a*, -*7d*, and -*7g* by binding to their promoters, thus, forming a negative-feedback loop (Chang et al., 2008; Wang et al., 2011).

The involvement of *let-7* miRNA in stem cell regulation also provided a clue as to how *let-7* may function as a tumor suppressor. *let-7* was shown to regulate the expression of high-mobility group AT-hook 2 (HMGA2), which is an early embryonic oncofetal gene that is overexpressed in stem cells and contributes to their self-renewal (Yu et al., 2007; Nishino et al., 2008). Thus, one of the mechanisms of maintaining undifferentiated state in stem cells is upregulation of HMGA2 by maintaining the low level of *let-7* miRNA. During differentiation, increased expression of *let-7* down-regulates HMGA2 by interacting with its 3′ UTR (Yu et al., 2007; Boyerinas et al., 2008; Nishino et al., 2008). The inverse relationship between the expression levels of *let-7* and HMGA2 was further supported by recent studies demonstrating that ectopic *let-7* expression can inhibit cell growth and mammosphere formation by down-regulating RAS and HMGA2 in mouse breast cancers (Sempere et al., 2007; Yu et al., 2007). Together, these lines of evidence strongly suggest that the *let-7* family members act as crucial tumor suppressors that inhibit diverse oncogenes.

In summary, two major biological roles have been elucidated for the *let-7* miRNA: as an essential regulator of terminal differentiation, and as a fundamental tumor suppressor. It thus seems that *let-7* should be expressed at specific stages of terminal differentiation, but down-regulated in stem cells being maintained in their undifferentiated state.

## PATHWAYS OF miRNA BIOGENESIS

### Canonical miRNA biogenesis pathway

**Figure 3:**
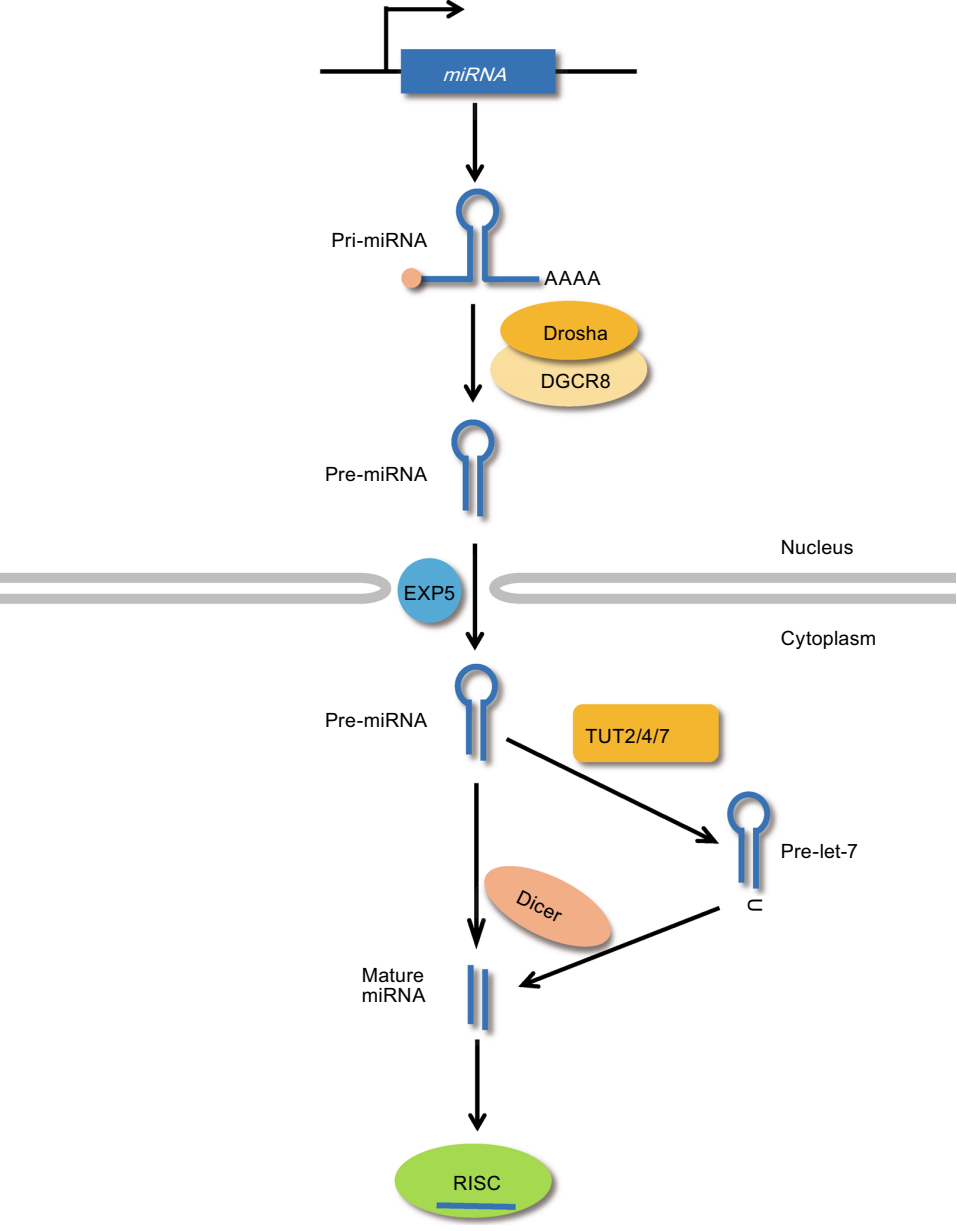
**Canonical pathway of miRNA biogenesis**. Schematic diagram of the canonical miRNA biogenesis process. A primary miRNA transcript produced by RNA polymerase II is processed by the Drosha microprocessor in the nucleus. The generated pre-miRNA is transported to the cytoplasm in an EXP5-Ran-GTP-dependent manner and further processed by the Dicer microprocessor to generate a mature miRNA. *Pre-let-7* is mono-uridylated at the 3′ end by LIN28A and TUTases prior to Dicer-mediated processing. The mature miRNA is loaded onto RISC to inhibit the translation of a target mRNA

The canonical miRNA biogenesis pathway is dependent on two microprocessors: Drosha and Dicer (Fig. 3). RNA polymerase II produces a primary miRNA transcript with a 5′ cap and a 3′ poly(A) tail from the encoding genomic locus (Bracht et al., 2004). Internal base-pairing within the primary miRNA (pri-miRNA) forms a characteristic hairpin stem-loop structure with a stem of ~33 bp in length. The pri-miRNA is subsequently processed by a microprocessor complex composed of the RNase III enzyme, Drosha, and the double-stranded RNA binding protein, DiGeorge syndrome critical region 8 (DGCR8; also known as Pasha), which cleaves the stem-loop structure into a 60–70-nt-long pre-miRNA that has a two-nt-long 3′ overhang (Lee et al., 2003; Denli et al., 2004; Gregory et al., 2004; Landthaler et al., 2004). The Drosha/DGCR8 microprocessor is a heterotrimeric complex consisting of one Drosha and two DGCR8 proteins. Following its processing by this Drosha/DGCR8 complex, the pre-miRNA is exported from the nucleus to the cytoplasm by the Ran-GTP-dependent transporter, exportin 5 (EXP5). When the pre-miRNA/EXP5/Ran-GTP complex is exported to the cytoplasm through the nuclear pore complex, GTP is hydrolyzed and the pre-miRNA subsequently dissociates (Yi et al., 2003; Bohnsack et al., 2004; Lund et al., 2004).

Following its transport into the cytoplasm, the pre-miRNA is further processed by Dicer into an RNA duplex of ~22 bp (Bernstein et al., 2001; Grishok et al., 2001; Hutvagner et al., 2001; Ketting et al., 2001; Knight and Bass, 2001). Dicer cleaves the pre-miRNA at a fixed length away from the base of the stem-loop, removing the loop to produce the 22-bp RNA duplex (Zhang et al., 2002; Zhang et al., 2004; Vermeulen et al., 2005; Macrae et al., 2006; MacRae et al., 2007; Park et al., 2011). Dicer may act together with transactivation response RNA-binding protein (TRBP) or protein activator of PKR (PACT; also known as PRKRA) in mammals (Lee et al., 2006; Lee et al., 2013a). These cofactors are dsRNA-binding proteins that have differential preferences for siRNA and miRNA. TRBP recruits Argonaute (AGO); however, the exact role of TRBP and PACT in miRNA biogenesis have not yet been fully elucidated.

One strand of the small dsRNA processed by Dicer, called a guide strand, is loaded onto an AGO protein to form RISC, which recognizes a target sequence that is usually embedded within the 3′ UTR region of a target mRNA in the P-body (Gregory et al., 2005; Liu et al., 2005; Eulalio et al., 2007). The *Drosophila* expresses two AGO proteins: AGO1, which preferentially associates with miRNAs, and AGO2, which binds to siRNAs (Okamura et al., 2004). The human has four AGO proteins; all of them have affinities for both siRNAs and miRNAs, and there does not appear to be any sorting mechanism to distinguish between siRNAs and miRNAs (Liu et al., 2004; Meister et al., 2004; Azuma-Mukai et al., 2008; Su et al., 2009; Dueck et al., 2012). RISC-incorporated mature miRNAs can block gene expression via a post-transcriptional mechanism, such as by inhibiting translation or facilitating mRNA degradation (Eulalio et al., 2008; Filipowicz et al., 2008).

Although *let-7* maturation generally follows the canonical miRNA biogenesis pathway, some family members require an additional step. Three members of the *let-7* family (*pre*-*let-7a-2*, -7c, and -7*e*) carry the typical two-nucleotide 3′ overhang in their precursors (group I pre-miRNAs), while the rest possess one-nucleotide 3′ overhang (group II pre-miRNAs) (Heo et al., 2012). The group II *pri*-*let-7* precursors have a bulged adenosine (*pri-let-7d*) or uridine (all other members of the group) next to the processing site (Heo et al., 2012). Drosha may fail to recognize this uridine/adenosine bulge, resulting in the generation of a one-nucleotide 3′ overhang. Due to this structural difference, an additional step is required to ensure efficient Dicer activity during biogenesis (Heo et al., 2012). In this step, terminal uridylyl transferases (TUT2/PAPD4/GLD2, TUT4/ZCCHC11, and TUT7/ZCCHC6) specifically mono-uridylate the 3′ end of the group II *pre-let-7*s, yielding the two-nucleotide 3′ overhang preferred by Dicer (Heo et al., 2012).

### The non-canonical miRNA biogenesis pathway

Although *let-7* family is generated through canonical miRNA biogenesis pathway, it would be helpful to understand the *let-7* biogenesis when comparing with the non-canonical miRNA biogenesis. The non-canonical miRNA pathways are well summarized in recent reviews (Ameres and Zamore, 2013; Ha and Kim, 2014). While the canonical miRNA biogenesis pathway depends on Drosha and Dicer, a small subset of miRNAs is processed independent of Drosha or Dicer. The biogenesis of mirtron, which was the first non-canonical biogenesis pathway to be discovered, is a Drosha-independent pathway (Berezikov et al., 2007; Okamura et al., 2007; Ruby et al., 2007). Mirtrons are encoded in an intronic region, such that the precursor is generated through an mRNA splicing mechanism that does not require Drosha. After splicing, lariat debranching and refolding converts the lariat to a pre-miRNA-like structure that is then subjected to Dicer cleavage (Okamura et al., 2007; Ruby et al., 2007). Mirtrons that contain additional sequences at their 5′ or 3′ ends are further trimmed by an exonuclease (Flynt et al., 2010). After trimming, the mirtrons can be processed by Dicer in a manner similar to that seen in the canonical miRNA pathway.

A Dicer-independent biogenesis pathway was also recently reported for a miRNA, as the maturation of *miR-451* was shown to require Drosha but not Dicer (Cheloufi et al., 2010; Cifuentes et al., 2010; Yang et al., 2010). Drosha-dependently processed *pre-miR-451* has a stem of only ~18 bp, which is too short for Dicer-mediated cleavage. Instead, *pre-miR-451* is directly loaded onto RISC, where AGO2-dependent cleavage generates *ac-pre-miR-451* (AGO-cleaved *pre-miR-451*) (Cheloufi et al., 2010; Cifuentes et al., 2010; Yang et al., 2010). Thereafter, the poly(A)-specific ribonuclease (PARN) further trims the 3′ end of *ac-pre-miR-451* to generate mature versions of *miR-451* harboring divergent 3′ ends (Yoda et al., 2013).

## REGULATION OF *let-7* BIOGENESIS

Dysregulation of *let-7* family members leads to abnormal physiological processes. The *let-7* mutant is lethal in the nematode (Reinhart et al., 2000), and decreased *let-7* expression or genomic deletion has been detected in several human cancer types (Takamizawa et al., 2004; Dahiya et al., 2008; O’Hara et al., 2009). In addition, while the mature *let-7* miRNA is not detected, *pri-let-7* exists in some cell types including mESCs (Suh et al., 2004; Thomson et al., 2006; Wulczyn et al., 2007). The observation that *let-7* expression gradually increases during development suggests that *let-7* biogenesis may be tightly regulated by additional factors (Pasquinelli et al., 2000; Sempere et al., 2002; Thomson et al., 2006; Liu et al., 2007). To date, several transcriptional and post-transcriptional mechanisms have been proposed as regulators of *let-7* biogenesis.

### Transcriptional regulation of *let-7*

*C. elegans* harbors a feedback circuit between *let-7* and the nuclear hormone receptor, DAF-12, in that DAF-12 is a target of *let-7*, but also regulates the transcription of *let-7* in a ligand-dependent manner. In an unfavorable environment, ligand-unbound DAF-12 suppresses *let-7* expression with its co-repressor, DIN-1. When environmental conditions favor developmental progression, however, ligand-bound DAF-12 activates the transcription of *let-7*. This feedback loop may regulate cellular fate and developmental arrest (Bethke et al., 2009; Hammell et al., 2009). Interestingly, a similar feedback loop has also been demonstrated in mammals: MYC is a target of *let-7*, but it can also repress the transcription of *let-7* during MYC-mediated tumorigenesis by directly binding to the promoter and upstream region of the *let-7a-1/let-7f-1/let-7d* cluster (Chang et al., 2008; Wang et al., 2011). Consistent with this idea of a negative feedback loop, shRNA-mediated suppression of endogenous MYC was found to up-regulate *let-7* (Wang et al., 2011), whereas *let-7* expression was shown to suppress MYC expression in a Burkitt lymphoma cell line (Sampson et al., 2007). Based on this, it seems reasonable to speculate that other transcription factors may also participate in the transcriptional regulation of *let-7* family members.

Even though *let-7* is ubiquitously expressed in adult mammalian tissues (Sempere et al., 2004), expression of individual *let-7* family members is also context-dependent. For example, *let-7i* is relatively enriched in thyroid compared to the other tissues (Lee et al., 2008). In addition, a subset of *let-7* family member would be expressed in specific tissues, cell lines, and cancers (Boyerinas et al., 2010; Chiu et al., 2014). This context-dependent expression of *let-7* family members would be tightly related with the expression of LIN28A/B as well as transcription factors (Thornton and Gregory, 2012). Despite *let-7* is one of the first discovered miRNAs, the details on transcriptional regulation of *let-7* family, especially individual members of *let-7* family, are not clearly understood. For this reason, mechanistic studies of transcriptional regulation should be further determined.

### Oligo-uridylation by TUTases is a marker for *pre-let-7* degradation

**Figure 4:**
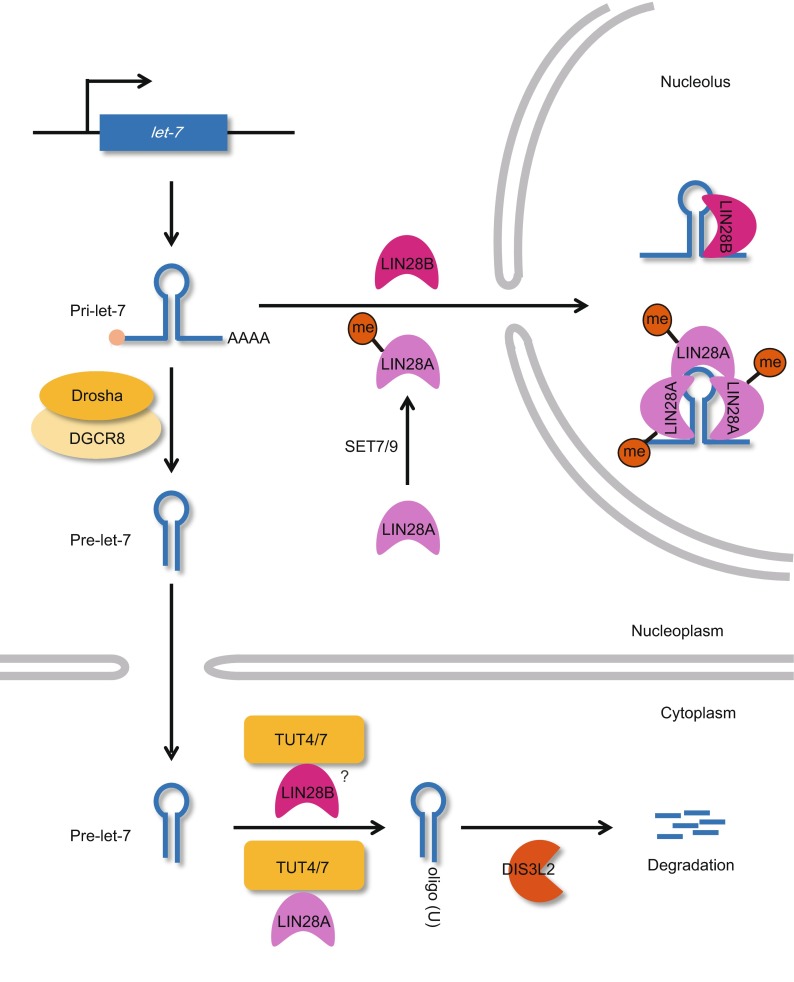
**Regulation of** ***let-7*** **biogenesis by LIN28A/B**. LIN28A and LIN28B inhibit the biogenesis of *let-7* via both TUTase-dependent and -independent pathways. LIN28A helps TUTases to oligo-uridylate *pre-let-7*. Methylated LIN28A binds to *pri-let-7* in the nucleus and sequesters it into the nucleolus to prevent Drosha-mediated processing. LIN28B blocks the biogenesis of the *let-7* miRNA via TUTase-independent pathways. The detailed relationship between LIN28B and TUTases needs to be further understood

It has been reported that *let-7* is also post-transcriptionally regulated by additional factors. As discussed above, TUTase is essential for the processing of the group II *pre-let-7* miRNAs, which have a unique 3′ overhang (Fig. 3) (Heo et al., 2012). Interestingly, the TUTases play a second role in the degradation of *pre*-*let-7* through their terminal uridylation activity (Fig. 4) (Heo et al., 2008; Hagan et al., 2009; Heo et al., 2009; Thornton et al., 2012). When LIN28A is overexpressed in HEK293T cells, the 3′-terminal oligo-uridylation of *pre*-*let-7* yields a uridine tail of ~14 nt (Heo et al., 2008). This oligo-uridylated *pre-let-7* resists Dicer cleavage and is instead susceptible to degradation. TUT4 and TUT7 were recently shown to oligo-uridylate *pre-let-7* in embryonic stem cells and cancer cells (Hagan et al., 2009; Heo et al., 2009; Thornton et al., 2012). The machinery responsible for degrading oligo-uridylated *pre-let-7* was recently identified as the catalytic subunit of the cytoplasmic exosome, DIS3L2 (Chang et al., 2013; Malecki et al., 2013; Ustianenko et al., 2013). The activity of DIS3L2 is stimulated when the uridine tail is at least 10 nt long, and it shows maximal activity against tails of 14 nt or longer. X-ray crystallography has shown that the three RNA binding domains of DIS3L2 form an open funnel that facilitates uridine-specific interactions with the first 12 uridines of the *pre*-*let-7* tail. This structural feature forms the basis for the substrate specificity of DIS3L2 (Faehnle et al., 2014).

### LIN28A/B negatively regulates *let-7* biogenesis

As noted above, LIN28A is required for the oligo-uridylation of *pre-let-7* by TUTases (Heo et al., 2008; Hagan et al., 2009; Heo et al., 2009; Piskounova et al., 2011; Thornton et al., 2012). LIN28, which was originally identified as a heterochronic gene in *C. elegans*, is evolutionarily conserved in animals. Mammals have two paralogs of LIN28, LIN28A (also known as LIN28) and LIN28B, which can bind to both *pri-* and *pre-let-7* to block the activities of Drosha and Dicer (Fig. 4) (Heo et al., 2008; Newman et al., 2008; Rybak et al., 2008; Viswanathan et al., 2008). LIN28A and LIN28B each have two RNA-binding domains, a cold-shock domain and a zinc finger motif (Moss and Tang, 2003). Through its RNA-binding activity, LIN28A associates with the bulging GGAG motif in the terminal loop of *pre-let-7* and recruits TUT4/7 (Nam et al., 2011). The terminal loop of *pre*-*let-7* has three independent binding sites for LIN28A, which can be multiply assembled in a stepwise fashion (Desjardins et al., 2014). This multimerization of LIN28A is likely to be required for the efficient blockade of Dicer-dependent *pre-let-7* processing. LIN28A reportedly competes with Dicer for *pre-let-7* and blocks processing of the precursor (Rybak et al., 2008); in the absence of LIN28A, *pre-let-7* is mono-uridylated by TUT2/4/7 and further processed by Dicer to generate the mature *let-7* (Heo et al., 2012). Thus, LIN28A blocks the Dicer activity in the cytoplasm, which is a TUTase-dependent pathway.

Interestingly, LIN28A also blocks Drosha-mediated processing in the nucleus (Newman et al., 2008; Viswanathan et al., 2008). Purified LIN28A inhibits *pri-let-7* processing *in vitro* and its ectopic expression selectively blocks *pri-let-7* processing *in vivo* (Newman et al., 2008; Viswanathan et al., 2008). In addition, *pri-let-7* processing is rescued by knockdown of LIN28A in mouse embryonal carcinoma (Viswanathan et al., 2008). Thus, although it is not yet clear whether LIN28A directly inhibits Drosha activity, it appears to negatively regulate *let-7* biogenesis in the nucleus as well as in the cytoplasm. LIN28A is mainly localized in the cytoplasm, but it can enter the nucleus and shows affinity for both *pri-* and *pre-let-7* (Heo et al., 2008; Newman et al., 2008; Rybak et al., 2008; Viswanathan et al., 2008). These lines of evidence suggest that LIN28A might participate in multiple steps of *let-7* biogenesis, including both Dicer- and Drosha-mediated processing.

LIN28B has also been shown to inhibit *let-7* biogenesis (Fig. 4), but the similar functions of LIN28A and LIN28B are achieved through very different action mechanisms (Piskounova et al., 2011). LIN28B was originally reported to have no affinity for TUTases, and the expressions of LIN28A and LIN28B appear to be mutually exclusive (Piskounova et al., 2011). In addition, LIN28B has a NoLS (nucleolar-localization sequence), and thus could be localized in the nucleolus. LIN28B appears to directly bind to *pri-let-7* in the nucleus and sequester it to the nucleolus, which lacks Drosha, thereby suppressing *let-7* maturation via a TUTase-independent pathway. Interestingly, however, a recent study showed that LIN28B interacts with DIS3L2 in the cytoplasm of LIN28B-expressing cancer cell lines, indicating that it also participates in the TUTase-dependent pathway (Suzuki et al., 2015). In this context, the level of *pre-let-7* appears to influence the subcellular localization of LIN28B (Suzuki et al., 2015).

### Post-translational modification changes the action mode of LIN28A

It was recently shown that LIN28A can prevent the biogenesis of *let-7* independent of TUT4/7 in hESCs, in a manner similar to that seen for LIN28B (Fig. 4) (Kim et al., 2014). The histone H3K4 methyltransferase, SET7/9, can mono-methylate LIN28A at lysine 135, which is near a sequence that is homologous to the NoLS of LIN28B (Kim et al., 2014). This sequence might be required for the nuclear (and especially nucleolar) localization of methylated LIN28A, which is its nuclear form. Electrophoretic mobility shift assays (EMSAs) have shown that the nuclear form of LIN28A binds to *pri-let-7* in a stepwise manner similar to its multimerization with *pre-let-7* (Desjardins et al., 2014; Kim et al., 2014). In addition, methylated LIN28A has a higher binding affinity for *pri-let-7* compared to cytoplasmic unmethylated LIN28A, whereas the affinity for *pre-let-7* does not differ between the two (Kim et al., 2014). Thus, it appears that LIN28A may regulate *pri-let-7* processing in a TUTase-independent fashion in the nucleus as well as a TUTase-dependent pathway in the cytoplasm. Moreover, the SET7/9-mediated post-translational modification (methylation) appears to act as a switch that changes the action mode of LIN28A in the inhibition of *let-7* biogenesis.

## SUMMARY AND PERSPECTIVES

In this review, we provide an overview of the features and biological roles of the *let-7* family members in higher eukaryotes. As *let-7* is induced during development and represses the expression of pluripotency factors, its biogenesis must be precisely regulated. In general, the *let-7* miRNA is generated through the canonical miRNA biogenesis pathway, which involves Drosha- and Dicer-dependent processing and is supported by TUTases. In the presence of LIN28A/B, TUTases instead inhibit *pre-let-7* processing by oligo-uridylation via LIN28A/B-mediated targeting. LIN28A/B proteins also regulate *let-7* biogenesis via TUTase-independent pathways. In the case of LIN28A, methylation seems to act as a switch, changing both its subcellular localization and its action mechanism. Although the expressions of LIN28A and LIN28B are mutually exclusive and these proteins play somewhat different inhibitory roles in *let-7* biogenesis, recent results suggest that they might share the consensus of their molecular mechanism. Indeed, compensatory redundancy between LIN28A and LIN28B has been observed (Wilbert et al., 2012).

At present, the detailed molecular mechanisms underlying *let-7* miRNA biogenesis are not fully understood. For instance, we do not yet know what happens to *pri-let-7* following its sequestration into the nucleolus by methylated LIN28A or LIN28B. The details of the relationship between DIS3L2-related cytoplasmic exosomes and *let-7* biogenesis are also unknown. Indeed, DIS3, other catalytic subunit of cytoplasmic exosome, also indirectly regulates the expression of *let-7* through degradation of LIN28B mRNAs in several mammalian cancer cell lines (Segalla et al., 2015). Emerging evidence suggests that the activities of the regulatory machineries are likely to be fine-tuned by post-translational modifications. In fact, the deacetylation of DGCR8 by HDAC1 was shown to increase the affinity for pri-miRNAs (Wada et al., 2012). Further studies examining the molecular mechanisms of *let-7* biogenesis and its regulation by nuclear/nucleolar and cytoplasmic factors should provide new insights into the biological roles of the *let-7* family members. Ultimately, detailed mechanistic studies for *let-7* biogenesis and its regulation involved in the developmental timing, cell division and differentiation in animals should be elucidated.

## ABBREVIATIONS

EMSAs: electrophoretic mobility shift assays
HMGA2: high-mobility group AT-hook 2
HSPC: hematopoietic stem and progenitor cell
*Let-7*: *lethal-7*
miRNAs: microRNAs
NSCLC: non-small cell lung cancer
PARN: poly(A)-specific ribonuclease
*pre-let-7*: *let-7* precursor
RISC: RNA-induced silencing complex
